# Phytochemical Constituent Analysis of *Phyllanthus emblica* L. Fruit Nanoherbals by LC-HRMS and Their Antimutagenic Activity and Teratogenic Effects

**DOI:** 10.3390/molecules29071642

**Published:** 2024-04-06

**Authors:** Masfria, Aminah Dalimunthe, Nurul Suci, Hafid Syahputra

**Affiliations:** 1 Department of Pharmaceutical Chemistry, Faculty of Pharmacy, Universitas Sumatera Utara, Medan 20155, Indonesia; masfria@usu.ac.id; 2 Nanomedicine Center of Innovation, Universitas Sumatera Utara, Medan 20155, Indonesia; 3 Department of Pharmacology, Faculty of Pharmacy, Universitas Sumatera Utara, Medan 20155, Indonesia; aminah@usu.ac.id; 4 Department of Pharmaceutics and Pharmaceutical Technology, Faculty of Pharmacy, Universitas Sumatera Utara, Medan 20155, Indonesia; nurulsuci@usu.ac.id

**Keywords:** *Phyllanthus emblica* L. fruit nanoherbal, antimutagenic, teratogenic, LC-HRMS

## Abstract

Pregnant women must be wary of using traditional medicines due to the possibility of their having oxytoxic effects. Indonesia is rich in plants containing antioxidants. One of these plants is *Phyllanthus emblica* L. This study aims to determine the phytochemical constituents of *Phyllanthus emblica* L. fruit nanoherbals by LC-HRMS analysis and their antimutagenic activity and teratogenic effects. The study commenced by producing nanoherbal extracts from *P. emblica* fruit. The phytochemical composition of these extracts was then analyzed using LC-HRMS. The nanoherbal extracts were also tested for their ability to prevent mutations, as indicated by a reduction in micronuclei observed in mouse femur bone marrow smear preparations. The teratogenicity test involved administering the *P. emblica* fruit nanoherbal at 100, 500, and 1000 mg/kg BW doses. The data were analyzed using SPSS. The phytochemical constituents of the *P. emblica* fruit nanoherbal include flavonoids, phenols, vitamins, and alkaloids. The *P. emblica* fruit nanoherbal exhibits antimutagenic activity, as evidenced by a statistical analysis that indicated a significant decrease in the quantity of micronuclei per 200 PCE compared to the negative control (*p* < 0.05). The administration of the *P. emblica* fruit nanoherbal at a dosage of 1000 mg/kg BW resulted in a teratogenic impact during the organogenesis stage, as shown by hemorrhage and anomalies in the sternum.

## 1. Introduction

Research on the consumption of traditional medicines and their effects on fetuses has not been clinically proven. However, research conducted on experimental animals shows that some medicinal plants used as herbal medicine for pregnant women are oxytocic (stimulate the uterus), causing uterine and intestinal bleeding, fetal death, and abnormal (slow) fetal growth. Therefore, pregnant women should be wary of using traditional medicines. Pregnant women often consume certain natural ingredients of traditional herbal medicines, which have an oxytoxic effect that compromises the fetus’s safety in the womb [1].

Medicinal plants often used by the community must be tested for their potential toxic effects to ensure the safety of the plants when used. A toxicity test is a form of examination that can be conducted to ascertain the safety of medicinal herbs. A toxicity test is conducted to identify the harmful impact of a drug on a living organism and to gather standard dose–response information from the test sample. The acquired data can be utilized to ascertain the risk associated with the test preparation when people are exposed to it, hence enabling the determination of a safe dosage for human consumption [2].

A teratogenicity test is a specialized toxicity test that aims to analyze the specific impact of a drug on the developing fetus during pregnancy to determine the safety of using the substance in pregnant women. A teratogenicity test involves administering a test preparation at many dose levels to various groups of pregnant animals throughout the organogenesis period of pregnancy, with each group receiving a different dose [3]. The organogenesis stage during pregnancy is a crucial phase characterized by a highly intricate process of cellular differentiation, which leads to the formation of organs and tissues. Consequently, this period is highly vulnerable to the harmful effects of teratogenic drugs [4].

One of the fruits often consumed and utilized by Southeast Asian people is *P. emblica*. *P. emblica* belongs to the Phyllanthaceae family [5] and is a type of fruit native to Indonesia that grows wild in gardens and forests. This tree grows in Indonesia and is spread across the islands of Java, Sumatra, Kalimantan, Maluku, and Nusa Tenggara [6]. *P. emblica* has anti-inflammatory, antioxidant, antibacterial, antifungal, anticancer, and antidepressant properties and can treat other diseases. *P. emblica* fruit contains secondary metabolites, such as alkaloids, flavonoids, glycosides, tannins, saponins, steroids, and triterpenoids [7,8]. The potential contents of these secondary metabolites can have antimutagenic activity.

Mutations are changes in the nucleotide arrangement of DNA due to deletion, addition, transfer, or translocation. Therefore, if there is a change in the nucleotide arrangement of DNA, the amino acids that make up the protein that DNA codes for will change, so the protein in question will be abnormal. The abnormal protein will have an abnormal function [9]. An indicator of a mutation is the presence of a micronucleus. A micronucleus is formed when complete chromosomes undergo mutations and break, resulting in the appearance of tiny nuclei within a cell [10,11].

Therefore, *P. emblica* fruits must be tested for teratogenic toxicity and antimutagenic potential. Apart from that, direct use of natural ingredients is less practical because they have a large volume and are difficult to store and transport, so they need to be made in a more practical form, such as a nanoherbal [12]. The influence of drug particle size on dissolution rate and bioavailability is demonstrated comprehensively by drugs absorbed from the gastrointestinal tract. Reducing the particle size of a drug can generally increase its absorption rate and bioavailability. One effort to reduce particle size is the creation of nanoherbal forms [13].

Nanoherbals are herbs with particle sizes of around 1–1000 nm. Nanosizes have advantages compared to larger sizes. The smaller the particle size, the greater the surface area. A large surface area will cause a material to become more reactive. One of the advantages of nanoherbals is their potential to increase the release of secondary metabolites in tissues. The antimutagenic activity of rhaphidohora pinnata simplicia leaf extract and nanoparticles shows that the activity of simplicia nanoparticles is much stronger compared to that of ethanol extract because the absorption of simplicia nanoparticles in the intestine is greater due to their increased solubility and increased enterocyte membrane permeability and the opening of paracellular connections, which are tight between enterocytes [14,15].

Apart from these findings, research related to the synergistic mechanism of nanoparticles in the absorption process in the body shows that the pharmacological effects of isolated herbal compounds are sometimes weaker than those of simplicia/nanoherbal extracts. Therefore, there is an opinion that purer herbal extracts are sometimes less effective. In the pharmacokinetic process, there is a synergistic relationship between primary and secondary metabolites in plants, which increases the absorption of active substances in the intestine by increasing solubility. Therefore, there is a synergistic relationship between metabolites, and this is improved by forming them into nanoherbals, which help release metabolites better because the surface area is larger [16].

## 2. Results and Discussion

### 2.1. Phytochemical Constituent Analysis of P. emblica Fruit Nanoherbal by LC-HRMS

The *P. emblica* fruit nanoherbal underwent phytochemical constituent analysis using LC-HRMS to gather data on its chemical constituents. Table 1 displays the results; the *P. emblica* fruit nano-herbal contains several active compounds, such as flavonoids (quercetin and myricitin), phenols (kojic acid, ellagic acid, and coumaric acid), vitamins (nicotinamide, nicotinic acid, and choline), and alkaloids (trigonelline).

Phenol and flavonoid molecules can function as antioxidants by regulating natural antioxidants’ enzymatic and non-enzymatic action [17]. Some flavonoid compounds, like quercetin and myricitin, can lower nitric oxide levels, myeloperoxidase, and malondialdehyde (MDA). This makes them antioxidants [18]. Additionally, they can enhance the levels of non-enzymatic antioxidants like glutathione (GSH) and boost the activity of antioxidant enzymes such as catalase (CAT), glutathione peroxidase (GPX), and superoxide dismutase (SOD). Flavonoids have also been reported to protect cells from damage by modulating DNA repair, increasing broken DNA strand reunification rate [19].

Besides phenols and flavonoids, several other compounds also exhibit antioxidant activity. Kojic acid has been shown to reduce lipid peroxidation and reactive oxygen species in amyloid-beta (Aβ)-induced rat brains. Some antioxidant enzymes, like catalase (CAT), glutathione peroxidase (GPX), and superoxide dismutase (SOD), can be made more effectively by ellagic acid [18]. Coumaric acid can raise the levels of antioxidants that are not enzymes, like glutathione (GSH), and increase the activity of antioxidant enzymes, like catalase (CAT), glutathione peroxidase (GPX), and superoxide dismutase (SOD) [20]. Nicotinamide (1 g/kg BW intraperitoneally) increased levels of nicotinamide adenine dinucleotide (NAD). Nicotinamide, a precursor to NAD, protects against tertiary-butyl hydroperoxide-induced apoptosis [21,22]. Nicotinamide can also inhibit the initiation of lipid peroxidation [23]. Nicotinamide also has several benefits, such as the ability to increase levels of superoxidase dismutase (SOD), catalase (CAT), glutathione (GSH), and glutathione-s-transferase (GST) [24]. Nicotinic acid can increase levels of glutathione (GSH), catalase (CAT), and superoxidase dismutase (SOD) while reducing levels of malondialdehyde (MDA) [25]. Choline has been shown to enhance the production of superoxide dismutase (SOD), catalase (CAT), glutathione peroxidase (GPX), glutathione-S-transferase (GST), and glutathione reductase (GR) [26]. Trigonelline enhances the activity of superoxide dismutase (SOD), catalase (CAT), and glutathione peroxidase (GPX) [27]. The antioxidant activity of several compounds in the P. emblica fruit nanoherbal makes the *P. emblica* fruit nanoherbal have an antimutagenic effect.

The teratogenic effect is thought to be caused by the content of phenolic and flavonoid compounds contained in the *P. emblica* fruit nanoherbal. Phenol and flavonoid compounds have antioxidant activity but can act as pro-oxidants in certain circumstances. The pro-oxidant activity caused by flavonoids is concentration-dependent (depending on concentration) (at high concentrations of phenolic compounds and flavonoids); in the presence of metal ions and alkaline pH, phenolic compounds can become pro-oxidants [28]. This pro-oxidant activity is thought to induce lipid peroxidation, DNA damage, and apoptosis in normal and cancer cells [29]. Phenolic compounds and flavonoids with small molecular weights, such as gallic acid and quercetin, can show pro-oxidant activity [30]. In contrast, phenolic compounds and flavonoids with large molecular weights, such as tannins, have little or no pro-oxidant properties.

### 2.2. Antimutagenic Activity

**Figure 1:**
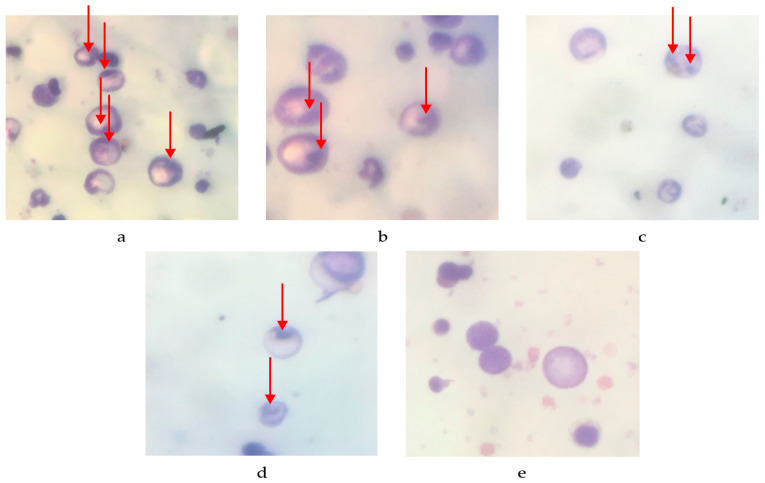
The cells observed in the mice femoral bone marrow smear. (**a**) Negative control; (**b**) 100 mg/kg BW; (**c**) 200 mg/kg BW; (**d**) 400 mg/kg BW; (**e**) normal control. (→: formed micronuclei).

The quantity of micronuclei present in the erythrocyte femur is displayed in Figure 1 and Table 2. There are 136.8 ± 6.591 micronuclei per 200 peripheral blood erythrocyte (PCE) cells in the negative control, 71.2 ± 3.611 in the 100 mg/kg BW dose, 47.6 ± 3.187 in the 200 mg/kg BW dose, 16.8 ± 2.059 in the normal control, and 0 ± 0 in the 400 mg/kg BW dose. The percentage reduction in the number of micronuclei per 200 PCE cells is as follows: 31.6% for the negative control, 64.4% for the 100 mg/kg BW dose, 76.2% for the 200 mg/kg BW dose, 91.6% for the 400 mg/kg BW treatment, and 100% for the normal control. These findings suggest a direct correlation between the dosage of *P. emblica* fruit nanoherbal and the reduction in micronucleus count.

This study used male mice to investigate *P. emblica* fruit nanoherbal's antimutagenicity using the micronucleus technique. The experimental mice were administered cyclophosphamide (50 mg/kg BW) via intra-peritoneal injection, which can stimulate the development of micronucleated cells. The antimutagenic activity was demonstrated by a reduction in the number of micronuclei observed per 200 poly-chromatic erythrocytes in the femoral bone marrow smear of mice.

The presence of a micronucleus serves as an indication of genetic mutation. Micronuclei are formed when intact chromosomes undergo mutations and become damaged, appearing as microscopic nuclei within a cell [31]. The micronucleus is small and round and located around the cytoplasm of erythrocyte cells. It has a size of approximately 1/20 to 1/5 of the nucleus of the cell [32].

In this study, micronucleus formation was induced by cyclophosphamide. Cyclophosphamide is an anticancer alkyl compound. Cyclophosphamide, as a mutagenic agent, damages bone marrow, interfering with the production of some or all forms of blood elements, which can cause anemia, leukopenia, and thrombocytopenia [33].

Cyclophosphamide induces micronucleus formation through active alkalizing metabolites: mustard phosphamide, acrolein, and 4-hydroxycyclophosphamide [34]. These alkalizing compounds can bind to various functional groups of cell components, including DNA bases. These bonds, among others, result in DNA crossover events and DNA chain breaks, which are thought to cause chromosomal breaks and can be seen as micronuclei. Cyclophosphamide metabolism has been reported to increase superoxide and hydroxyl anion radicals, which may play a role in inducing micronucleus formation [35].

The antimutagenic activity is related to compounds in the *P. emblica* fruit nanoherbal, namely phenolic compounds and flavonoids. Phenol and flavonoid compounds are responsible for the antioxidant activity of the *P. emblica* fruit nanoherbal. Some phenols and flavonoids in the *P. emblica* fruit nanoherbal are ascorbic acid, quercetin, geraniin, kaempferol, ellagic acid, and kojic acid [36]. A number of phenols, especially flavonoids, are antioxidant compounds that can overcome various kinds of free radicals. Because these flavonoids are antioxidants, they might help protect cells from damage caused by radicals like peroxide, hydroxyl, alkoxyl, and superoxide [37].

### 2.3. Teratogenic Effects

#### 2.3.1. Body Weight of Pregnant Rats

Body weight growth was seen from day 6 to day 19 of gestation following the P. emblica fruit nanoherbal intake. Table 3 displays the body weight of pregnant rats following treatment with different dosages of *P. emblica* fruit nanoherbal. Weight growth was found in all pregnant rats during gestation. According to the statistical analysis, there was no statistically significant difference (*p* > 0.05) in the average weight gain among all pregnant rats.

Rats were given the test solution during the organogenesis period, days 6 to 15 of gestation. From day 1 to day 5 of pregnancy, the mother rats were not given any treatment because, at that time, there was totipotency in the fetus, which could repair damaged tissue. Teratogenic chemicals caused functional impairments that were not detectable after birth on day 16 [38]. It is important to keep track of the mother rats’ body weight every day during organogenesis to see how well they eat while pregnant. Rats are particularly vulnerable to teratogenic chemicals during the organogenesis period of pregnancy, a crucial time due to the intense cell differentiation occurring during this period as organs and tissues form [39].

Maternal body weight during pregnancy increases fetal development and volume of amniotic fluid, placenta, and amniotic membranes [40]. During the observation, there were no mother rats that experienced spontaneous births. The results of the one-way ANOVA test on the observation of the average body weight of the parent during the organogenesis period showed no significant difference (*p* > 0.05) in the *P. emblica* fruit nanoherbal group at doses of 100 mg/kg BW, 500 mg/kg BW, 1000 mg/kg BW, and the negative control group was gabapentin 50 mg/kg BW with the control group CMC Na 0.5%.

#### 2.3.2. Litter Size

The fetuses were extracted from the uterus and thoroughly sanitized, after which the number of offspring in the litter was documented. According to the data presented in Table 4, there was no significant difference in the litter size across all the samples (*p* > 0.05). Every fetus remained viable after administering nanoherbal therapy derived from *P. emblica* fruit.

Fetal biometrics are quantitative data used to see the effect of the teratogen being tested. One of the fetal biometric data items includes the number of deaths, the number of live fetuses, and the number of dead fetuses.

The study findings indicate that the administration of *P. emblica* fruit nanoherbal did not result in fetal death or resorption. The mildest impact of a teratogenic drug [41,42] is observed in changes to litter size and body weight. The number of offspring produced and the weight of the mother’s body are indicators of the development of the fetus and the amount of nourishment provided to the fetus during pregnancy. Typically, the pregnant Wistar rats gave birth to litters containing 1–13 offspring, with 10 being the most common number. The litter size for normal pregnant rats in this study ranged from 9 to 11 fetuses, with a mean litter size of 10.20. The administration of the *P. emblica* fruit nanoherbal did not significantly impact litter size compared to the control group (*p* > 0.05).

#### 2.3.3. Litter Length and Birth Weight

**Figure 2:**
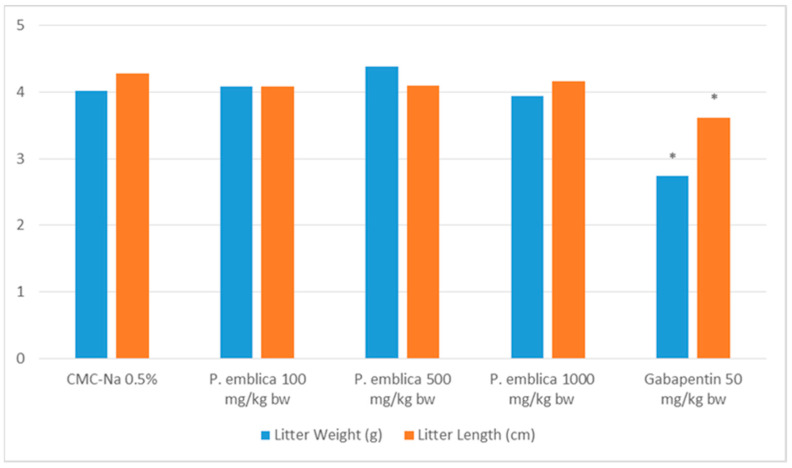
Effect of *P. emblica* nanoherbal treatment on litter length and birth weight (mean ± SEM, * *p* < 0.05 significant compared to control).

Assessing teratogenicity involves the evaluation of litter length and birth weight, which are both crucial characteristics. Figure 2 demonstrated that the *P. emblica* fruit nanoherbal had no significant impact on litter length and birth weight compared to the control group (*p* > 0.05).

#### 2.3.4. External Malformations

**Figure 3:**
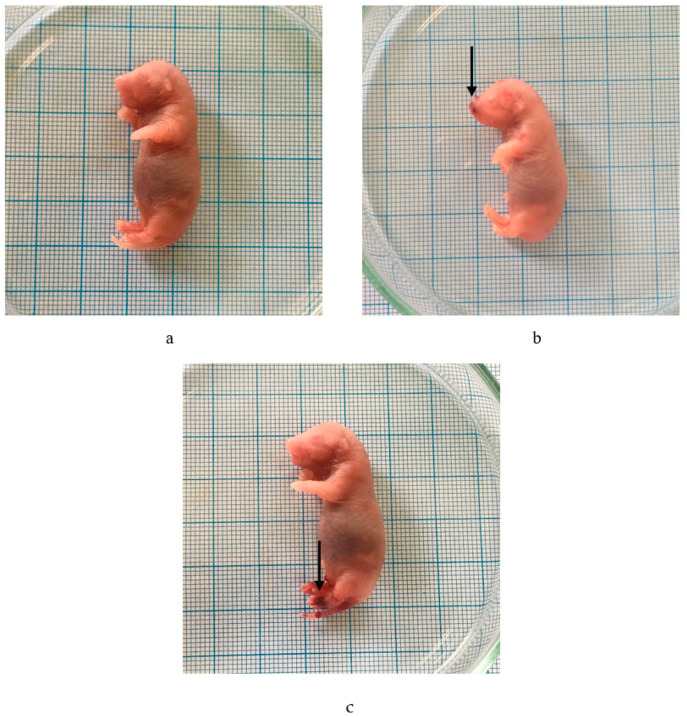
Effect of *P. emblica* fruit nanoherbal on external malformations. (**a**) Normal fetus; (**b**,**c**) fetus with hemorrhage. (→: hemorrhage).

Visible abnormalities were noticed on the outside of the fetus following its immersion in Bouin’s solution. Table 5 indicated that hemorrhage occurred following the administration of the *P. emblica* fruit nanoherbal at 1000 mg/kg BW (Table 5, Figure 3).

#### 2.3.5. Skeletal Malformations

**Figure 4:**
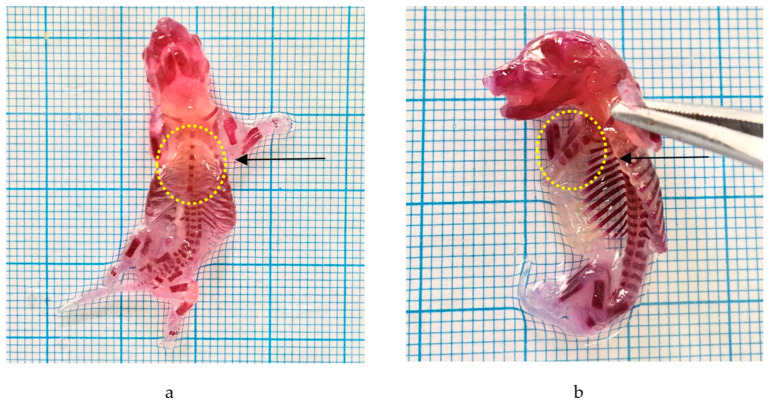
Effect of *P. emblica* fruit nanoherbal on external malformations. (**a**) Normal fetus; (**b**) fetus with abnormal sternum (5 sternums). (→: sternum).

Alizarin was employed to detect skeletal abnormalities. Administering the *P. emblica* fruit nanoherbal at a dosage of 1000 mg/kg BW resulted in skeletal deformities, namely an aberrant sternum. The data are shown in Table 6 and Figure 4.

Body weight is an important parameter in determining the effect of foreign compounds on the fetus. The fetus’s growth and development rate shows variations in litter size. Fetal body weight reduction is the most minimal form of teratogenic expression and is a more sensitive parameter for teratogenic testing [43]. The results of observations of fetal body weight and length showed that the *P. emblica* fruit nanoherbal did not affect the litter size compared to normal (*p* > 0.05).

The study of morphological abnormalities also showed that giving 1000 mg/kg BW of the P. emblica fruit nanoherbal caused deformities on both the skin and the bones.

Hemorrhage refers to the expulsion of blood from the circulatory system, accompanied by the buildup of blood in spaces or tissues within the body. This phenomenon arises when the extract is administered repeatedly at sufficiently high doses, leading to elevated bloodstream concentrations and an osmotic imbalance. Typically, the embryo grows in amniotic fluid with the same solute concentration as the bodily fluids. Exogenous chemicals present in tissues can alter the osmotic pressure. Osmotic imbalances can happen when there are changes in the viscosity and pressure of fluids in different parts of the embryo, such as the blood plasma, the extracapillary space, and the fluids inside and outside the embryo; hemorrhage results from bursting of the blood vessels due to this difference [44].

The *P. emblica* fruit nanoherbal at 1000 mg/kg BW caused six hemorrhagic fetuses. Inhibition of bone growth, especially ossification, can occur due to impaired calcium absorption by osteoblasts. Osteoprogenitor cells are bone cells active in dividing and forming osteoblasts that play a role in ossification. The induction of flavonoid compounds can interfere with cell replication and inhibit mitosis (cell division) at the metaphase stage by inhibiting the formation of the myolytic spindle so that it will cause chromosomes to break, spread, or clump due to dead cells [45].

It can also occur due to interference with calcium absorption by tannin compounds. Tannins can bind to proteins and cause a lack of protein absorbed by the parent body, interfering with osteoblast proliferation during bone formation. Tannins are compounds that can inhibit the absorption of nutrients in the intestine and increase the excretion of proteins and amino acids. The inhibition of absorption of these nutrients causes a lack of availability of nutrients the developing embryo needs (malnutrition). Malnutrition, especially lack of calcium, which the embryo needs during bone formation, can cause delays in ossification.

One of the determining factors in bone growth and development is hormones. The hormones that affect skeletal growth include GH, thyroxine, estrogen, and androgens. Growth hormone (GH) is produced from the pituitary gland, which functions to increase the mitotic process of chondrocytes and osteoblasts and the synthesis of collagen-forming proteins, cartilage matrix, and enzymes for cartilage and bone formation. Thyroxine is produced from the thyroid gland, which plays a role in increasing the process of protein synthesis. Estrogen functions in stimulating osteoblast activity and the process of bone matrix synthesis.

## 3. Materials and Methods

### 3.1. Materials

Fresh fruits of *P. emblica* were collected from South Tapanuli, North Sumatra, Indonesia. *P. emblica* was identified in Herbarium Medanense, Department of Biology, Faculty of Mathematics and Natural Sciences, Universitas Sumatera Utara, and the voucher specimen was deposited in an herbarium (No. 5581/MEDA/2021).

The fruits were transformed into simplicia powder and subsequently crushed at PT. Nanotech Herbal Indonesia is located in Bogor, Indonesia. The fruits were crushed top-down using high-speed milling to obtain particles with a size of 979.2 ± 237.7 nm. The Beckman Coulter Delsa nanoparticle instrument was used to measure the size of the particles at a temperature of 25 °C and with water as the particle solvent.

Chemicals that were used were methanol (Merck, Germany), Giemsa solution (Sigma-Adrich, Saint Louis, MO, USA), immersion oil, 0.9% NaCl (PT. Widatra Bhakti, Pasuruan, Indonesia), bovine blood serum, cyclophosphamide (Cyclovid®, PT Novell Pharmaceutical Laboratories, Jakarta, Indonesia), acetonitrile, distilled water (BrataChem, Jakarta, Indonesia), ethanol, ethylacetate, formic acid, Na-carboxymethylcellulose (Sigma, Livonia, MI, USA), and KOH (Merck, Darmstadt, Germany). The equipment utilized in this investigation consisted of surgical instruments (Spencer-Wells, San Francisco, CA, USA), a microscope (Boeco, BM 180, equipped with a halogen lamp, Hamburg, Germany), a centrifuge (Dynamica, model velocity 18R, Shenzhen, China), and microtubes.

### 3.2. Animals

The research was carried out on a cohort of twenty-five male mice, aged three months, weighing 20 and 30 g, and twenty-five female rats, also aged three months and weighing 180 and 200 g. These animals were sourced from the Animal House of the Faculty of Pharmacy at Universitas Sumatera Utara in Medan, Indonesia. They were kept in a good lab setting at room temperature (25 ± 2 °C), with 12 h of light and 12 h of darkness. They were fed standard pellets and could drink as much water as they wanted. The Ethics Committee of Animal Research of the Faculty of Mathematics and Natural Sciences, Universitas Sumatera Utara, authorized this research procedure. The approval number is 00206/KEPH FMIPA/2022.

### 3.3. Phytochemical Constituent Analysis of P. emblica Fruit Nanoherbal by LC-HRMS

Determination of the phytochemical constituents of the *P. emblica* fruit nanoherbal was carried out with UHPLC Thermo Scientific Dionex Ultimate 3000 RSLCnano—HRMS Thermo Scientific Q Exactive (LSIH, Brawijaya University) with mobile phase A (0.1% formic acid in water) and phase B (0.1% formic acid in acetonitrile) following the gradient method. A 40 μL/min flow rate was used for the Hypersil GOLD aQ column, 50 × 1 mm × 1.9 m. The column oven temperature was 30 °C, and the time for analysis was 30 min. The results were analyzed using Compound Discoverer software with mzCloud MS/MS Library 2.1 [46].

### 3.4. Antimutagenic Activity Testing

#### 3.4.1. Testing of Antimutagenic Effects in Mice

The mice were divided into five groups, each with five mice. The experimental design is shown in Table 7.

On the eighth day, the animal was sacrificed, and then the femur was taken and cleaned. The end of the proximal bone was cut, and then the femoral bone marrow was taken using a syringe containing 0.2 mL of bovine serum. The syringe was inserted into the open bone marrow channel to aspirate the marrow fluid so that it mixed with the bovine serum. After that, the mixture was put in a microtube [47,48,49].

#### 3.4.2. Preparation of Femoral Bone Marrow Smears

The combination of bone marrow and bovine serum in a microtube was subjected to centrifugation at a speed of 1200 revolutions per minute for a duration of 5 min, following which the liquid portion above the sediment was removed and discarded. The precipitate was reconstituted using two drops of bovine serum. Subsequently, a single droplet of cell suspension was extracted and deposited onto a slide. The droplet was then evenly distributed by dragging the substance beneath a smooth cover glass, which was positioned at a 45° angle. Subsequently, the slides were subjected to a drying process and treated with methanol for 5 min. Subsequently, the specimen was immersed in Giemsa solution for 10 min to facilitate staining. The slides were cleaned in distilled water, dried, and then examined under a microscope at a magnification of 10 × 100 using oil immersion. The count of micronucleus cells in 200 peripheral blood erythrocytes (PCEs) was determined.

### 3.5. Teratogenic Effect Testing

#### 3.5.1. Confirmation of Pregnancy

Three sexually receptive female rats were paired with two sexually active male rats in the afternoon and allowed to mate overnight in a cage. On the following day, the female rats were isolated from the male rats, and the vaginal smear was analyzed to assess pregnancy [50,51].

#### 3.5.2. Treatment

The pregnant rats were categorized into five groups, each including five pregnant rats. These include (1) normal group (received Na CMC 0.5%); (2) positive control (received gabapentin 50 mg/kg BW); (3) *P. emblica* fruit nanoherbal 100 mg/kg BW; (4) *P. emblica* fruit nanoherbal 500 mg/kg BW; (5) *P. emblica* fruit nanoherbal 1000 mg/kg BW.

Pregnant rats were orally administered Na CMC 0.5%, gabapentin, and a *P. emblica* fruit nanoherbal suspension in Na CMC 0.5% once daily, from day 6 to day 15 of gestation. Daily weights were taken on days 6–19 of pregnancy in rats. The rats were sacrificed on day 19, and information about the litter size, fetus deaths, and fetus resorption was logged. Two-thirds of each group's fetuses were used to look at external abnormalities, and one-third was used to look at skeletal malformations. Three days of soaking Bouin's fluid revealed the exterior abnormalities. The skeletal abnormalities were found after the bones were fixed with 96% for seven days and then 1% KOH for 12 h. Following the elimination of 1% KOH, alizarin was introduced, and the solution was saturated for 24 h. The skeletal malformations were observed under a microscope (10 × 40).

### 3.6. Statistical Analysis

The data were presented as mean ± SEM. All data were analyzed using one-way ANOVA and Tukey post hoc test.

## 4. Conclusions

There are phytochemical constituents, including flavonoids (quercetin and myricitin), phenols (kojic acid, ellagic acid, and coumaric acid), vitamins (nicotinamide, nicotinic acid, and choline), and alkaloids (trigonelline) in the *P. emblica* fruit nanoherbal based on the results of identification by LC-HRMS.

The *P. emblica* fruit nanoherbal at doses of 100, 200, and 400 mg/kg BW has antimutagenic activity by reducing the amount of micronuclei in PCEs. Increasing the dose of the *P. emblica* fruit nanoherbal increases antimutagenic activity.

The *P. emblica* fruit nanoherbal also had a teratogenic effect on the fetus during the organogenesis period, as indicated by the presence of hemorrhage and abnormalities in a number of sternums at a dose of 1000 mg/kg BW.

## Figures and Tables

**Table 1:** Secondary metabolite profile of *P. emblica* fruit nanoherbal.

No.	Name	Formula	Molecule Weight	Retention Time (min)
1	3,5-di-tert-Butyl-4-hydroxybenzoic acid	C_15_H_22_O_3_	250.15	15.02
2	NP-004917	C_15_H_26_O_3_	276.17	13.52
3	NP-020014	C_15_H_26_O_3_	276.17	17.57
4	2-[(2-chlorobenzyl)sulfanyl]-4,6-dimethylnicotinonitrile	C_15_H_13_ClN_2_S	326.00	4.63
5	3,5-di-tert-Butyl-4-hydroxybenzaldehyde	C_15_H_22_O_2_	234.16	17.04
6	Quercetin	C_15_H_10_O_7_	302.04	7.98
7	2-[(2-chlorobenzyl)sulfanyl]-4,6-dimethylnicotinonitrile	C_15_H_13_ClN_2_S	326.00	20.33
8	(2S,3R,4S,5S,6R)-3,4,5-trihydroxy-6-(hydroxymethyl)oxan-2-yl(2E)-3-phenylprop-2-enoate	C_15_H_18_O_7_	332.08	7.63
9	Kojic acid	C_6_H_6_O_4_	142.02	2.04
10	4-Coumaric acid	C_9_H_8_O_3_	164.04	2.06
11	Nicotinamide	C_6_H_6_N_2_O	122.04	2.05
12	Nicotinic acid	C_6_H_5_NO_2_	106.00	2.05
13	Ellagic acid	C_14_H_6_O_8_	302.00	7.19
14	3,4-Dihydroxyphenylpropionic acid	C_9_H_10_O_4_	164.04	13.51
15	Myricitrin	C_21_H_20_O_12_	464.09	7.14
16	Trigonelline	C_7_H_7_NO_2_	137.04	1.54
17	Betaine	C_5_H_11_NO_2_	117.07	1.51
18	Choline	C_5_H_13_NO	103.09	1.48
19	D-(+)-Proline	C_5_H_9_NO_2_	115.06	1.55

**Table 2:** The number of micronuclei in 200 polychromatic erythrocyte cells.

Group	Number of Micronucleus/200 Cells ± SEM	Percentage Reduction Micronucleus
Negative control	136.8 ± 6.591	31.6
Dose of 100 mg/kg BW	71.2 ± 3.611 *	64.4
Dose of 200 mg/kg BW	47.6 ± 3.187 *	76.2
Dose of 400 mg/kg BW	16.8 ± 2.059 *	91.6
Normal control	0 ± 0 *	100

* Significantly different from negative control. SEM: standard error of the mean.

**Table 3:** Body weight of pregnant rats after the treatment with *P. emblica* fruit nanoherbal.

Day	Body Weight (g) ± SEM (Mean ± SEM)				
	CMC Na 0.5%	*P. emblica* 100 mg/kg BW	*P. emblica* 500 mg/kg BW	*P. emblica* 1000 mg/kg BW	Gabapentin 50 mg/kg BW
6	217.10 ± 5.52	238.80 ± 7.99	232.84 ± 9.45	217.920 ± 7.02	216.46 ± 6.62
7	219.88 ± 5.91	240.64 ± 7.47	235.02 ± 9.45	219.48 ± 7.28	218.94 ± 6.77
8	222.30 ± 5.80	242.24 ± 7.60	235.30 ± 9.86	222.70 ± 6.82	221.64 ± 6.95
9	224.56 ± 5.76	243.86 ± 7.57	237.10 ± 9.94	224.80 ± 6.81	223.38 ± 6.80
10	228.06 ± 5.48	245.66 ± 7.55	239.08 ± 9.81	227.7 ± 6.81	224.32 ± 5.29
11	232.92 ± 6.01	247.54 ± 8.75	240.94 ± 9.69	228.34 ± 6.94	224.50 ± 6.54
12	238.78 ± 4.86	250.12 ± 8.42	242.76 ± 9.78	230.94 ± 7.03	227.50 ± 6.89
13	243.26 ± 4.53	251.26 ± 9.11	244.84 ± 9.71	232.86 ± 7.38	225.52 ± 5.84
14	250.26 ± 5.10	252.72 ± 9.04	251.46 ± 9.57	235.64 ± 7.23	227.82 ± 5.47
15	254.94 ± 4.62	255.90 ± 8.98	260.10 ± 9.69	239.34 ± 7.47	229.84 ± 5.52
16	261.14 ± 6.47	257.98 ± 8.97	269.06 ± 9.65	246.48 ± 6.10	248.04 ± 11.44
17	265.42 ± 7.16	260.08 ± 8.91	272.92 ± 9.67	255.48 ± 6.53	253.86 ± 12.04
18	268.78 ± 6.93	265.64 ± 9.41	274.92 ± 9.62	264.08 ± 7.07	262.42 ± 12.05
19	272.48 ± 8.31	267.66 ± 9.32	285.32 ± 9.68	274.96 ± 5.68	270.18 ± 10.22

**Table 4:** Litter size after the treatment with *P. emblica* fruit nanoherbal (mean ± SEM).

Samples	Number of Pregnant Rats	Litter Size
CMC-Na 0.5%	5	10.20 ± 0.37
*P. emblica* 100 mg/kg BW	5	9.40 ± 0.40
*P. emblica* 500 mg/kg BW	5	8.00 ± 1. 04
*P. emblica* 1000 mg/kg BW	5	9.80 ± 0.37
Gabapentin 50 mg/kg BW	5	9.80 ± 0.49

**Table 5:** Effect of *P. emblica* fruit nanoherbal on external malformations.

Parameters	Samples				
	CMC Na 0.5%	*P. emblica* 100 mg/kg BW	*P. emblica* 500 mg/kg BW	*P. emblica* 1000 mg/kg BW	Gabapentin 50 mg/kg BW
Number of fetuses examined	34	31	26	32	32
Stunted	-	-	-	-	8
Hemorrhage	-	-	-	6	8

**Table 6:** Effect of *P. emblica* fruit nanoherbal on skeletal malformations.

Parameters	Samples				
	CMC Na 0.5%	*P. emblica* 100 mg/kg BW	*P. emblica* 500 mg/kg BW	*P. emblica* 1000 mg/kg BW	Gabapentin 50 mg/kg BW
Number of fetuses examined	17	16	14	17	17
Truncus malformation					
*Sternum*	-	-	-	1	8
*Vertebrae caudals*	-	-	-	-	-

**Table 7:** Experimental Design.

Group	Treatment
I (negative control)	Animals were induced with 50 mg/kg BW cyclophosphamide solution on the first day, then administered CMC Na 0.5% suspension orally every day until the seventh day.
II (dose of 100 mg/kg BW)	In the test group, rats were induced with 50 mg/kg BW cyclophosphamide solution on the first day, then given 100 mg/kg BW *P. emblica* fruit nanoherbal orally every day until the seventh day.
III (dose of 200 mg/kg BW)	In the test group, rats were induced with 50 mg/kg BW cyclophosphamide solution on the first day, then given 200 mg/kg BW *P. emblica* fruit nanoherbal orally every day until the seventh day.
IV (dose of 400 mg/kg BW)	In the test group, rats were induced with 50 mg/kg BW cyclophosphamide solution on the first day, then given 400 mg/kg BW *P. emblica* fruit nanoherbal orally every day until the seventh day.
V (normal control)	Normal control animals received 0.5% CMC Na orally for 7 days.

CMC: Carboxymethyl cellulose, *P. emblica*: *Phyllantus emblica.*

## Data Availability

Data are contained within the article.
